# Children’s weight changes according to maternal perception of the child’s weight and health: A prospective cohort of Peruvian children

**DOI:** 10.1371/journal.pone.0175685

**Published:** 2017-04-19

**Authors:** Rodrigo M. Carrillo-Larco, Antonio Bernabe-Ortiz, J. Jaime Miranda, Hong Xue, Youfa Wang

**Affiliations:** 1 CRONICAS Centre of Excellence in Chronic Diseases, Universidad Peruana Cayetano Heredia, Lima, Peru; 2 Escuela de Medicina, Universidad Peruana de Ciencias Aplicadas, Lima, Peru; 3 Department of Medicine, School of Medicine, Universidad Peruana Cayetano Heredia, Lima, Peru; 4 Systems-Oriented Global Childhood Obesity Intervention Program, Department of Epidemiology and Environmental Health, School of Public Health and Health Professions, University at Buffalo, State University of New York, Buffalo, New York, United States of America; Laval University, CANADA

## Abstract

The aim of the study was to estimate the association between maternal perception of their child’s health status and (mis)classification of their child’s actual weight with future weight change. We present cross-sectional and longitudinal analyses from the Peruvian younger cohort of the Young Lives Study. For cross-sectional analysis, the exposure was maternal perception of child health status (better, same or worse); the outcome was underestimation or overestimation of the child’s actual weight. Mothers were asked about their perception of their child’s weight (same, lighter or heavier than other children). Actual weight status was defined with IOTF BMI cut-off points. For longitudinal analysis, the exposure was (mis)classification of the child’s actual weight; the outcome was the standardized mean difference between follow-up and baseline BMI. A Generalized Linear Model with Poisson family and log-link was used to report the prevalence ratio (PR) and 95% confidence intervals (95% CI) for cross-sectional analyses. A Linear Regression Model was used to report the longitudinal analysis as coefficient estimates (β) and 95% CI. Normal weight children who were perceived as more healthy than other children were more likely to have their weight overestimated (PR = 2.06); conversely, those who were perceived as less healthy than other children were more likely to have their weight underestimated (PR = 2.17). Mean follow-up time was 2.6 (SD: 0.3) years. Overall, underweight children whose weight was overestimated were more likely to gain BMI (β = 0.44); whilst overweight children whose weight was considered to be the same of their peers (β = -0.55), and those considered to be lighter than other children (β = -0.87), lost BMI. Maternal perception of the child’s health status seems to influence both overestimation and underestimation of the child’s actual weight status. Such weight (mis)perception may influence future BMI.

## Introduction

In the last three decades the prevalence of overweight and obesity has risen across all ages in developed and developing countries [1]. Current estimates for developing countries report a prevalence rate of overweight and obesity in children to be approximately 13% [1]. In Latin America, there are between twenty-two and twenty-five million overweight or obese school-aged children [2]. Peru has also seen an increase in the number of overweight children: the prevalence of overweight children (6 to 9 years old) ranges between 10.2% and 12.1% [3]. Although there are some effective methods to manage childhood overweight [4,5], parental participation and engagement are necessary for their success. Parents may refuse participation in weight loss strategies because they may not acknowledge the health risks associated with obesity [6,7], or they may not consider their child to be overweight or obese [7–11].

The literature shows that parents do not accurately identify their overweight children [6,8,12–15]. Such (mis)perception may depend on cultural and ethnic factors [16–19]. Mexican mothers living in Mexico, compared to Mexican mothers living in the USA, want their children to be bigger [18]. In addition, foreign-born Hispanic mothers with fewer years in the USA do not associate childhood obesity with poor health. This suggests a biased view that heavier equals healthier [17,20–24], signaling that mother’s perception of their child’s health could influence how they subjectively assess their child’s weight status.

However, many studies about (mis)perception of a child’s weight status were conducted in developed countries. This accounts for the need to further explore the (in)accurate perception of a child’s weight status in developing countries where cultural identity and ethnicity influence weight and health perception [17,20–24]. Moreover, many studies followed a cross-sectional design, leaving a knowledge gap on how parental (mis)perception influences weight changes [25]. Furthermore, how maternal perception of their child’s health influences their subjective assessment of their child’s weight status has been understudied; also, if these features influences weight changes over time has not been addressed.

Using prospective data from Peruvian children we aimed to: i) assess the agreement between maternal perception of their child’s weight status and actual weight status; ii) to assess if maternal perception of their child’s health status is a determinant of underestimation or overestimation of actual child weight status; and iii) to assess if underestimation or overestimation of actual child weight status influences future child weight according to maternal perception of their child’s health status.

## Materials and methods

### Study design and data source

Data of the Young Lives Study were analyzed. The Young Lives Study is a prospective cohort conducted in four developing countries: Ethiopia, India, Peru and Vietnam. The study started in 2002 with a younger and older cohort. The younger cohort enrolled at baseline approximately 2,000 children aged 6–18 months, while the older cohort enrolled approximately 1,000 children aged 7–8 years old. Follow-up rounds were conducted in 2006 and 2009. Further details about the Young Lives Study have been published elsewhere [26,27]. In brief, it is an international prospective cohort aiming to understand how childhood poverty changes in the study settings, and what effects it has on children´s nutrition, health, well-being, education, cognitive and physical development, among other parameters. Qualitative and quantitative methods were conducted.

### Participants

We used data of the second (hereafter known as *baseline*) and third (hereafter known as *follow-up*) rounds of the younger Peruvian Cohort. We did not include the baseline assessment because we targeted children not infants. Subjects with missing data on the following variables were excluded from the analysis: child age, sex and BMI, and maternal perception of child weight. We only included Peru as preliminary analyses showed this country had a higher prevalence of overweight children. In addition, because the authors are Peruvian, we felt confident to have a better understanding of the results than if we had included other countries; lastly, because of slightly differences in sampling methods, although it could be argued that merging all countries is appropriate.

The initial sample frame, including both rural and urban settings, was at the district level from which twenty sentinel sites were selected. To choose the sentinel sites a multi-stage, cluster-stratified, random sampling technique was followed. Afterwards, one census track in each district was randomly selected, and all block of houses and cluster of houses were counted; in each selected block or cluster, households were searched seeking those with at least one child in the age range. The sampling technique excluded the 5% of the richest districts according to poverty levels set by the Peruvian National Fund for Development and Social Compensation [28].

### Variables

#### Outcome variables

For the cross-sectional analysis, the outcome was overestimation or underestimation of actual child weight status. To define this variable we used maternal perception of their child’s weight and the actual child’s weight. We used three categories to define actual weight status: underweight, normal weight, and overweight (includes obesity) based on BMI with sex- and age-specific cut-off points [29]. Maternal perception was assessed with a questionnaire using the following question: *Compared to other children of this age would you say ‘NAME’s’ weight is the same*, *heavier or lighter*? Underestimation of actual weight status was defined as seeing the child at any lower weight status than he/she really is, for example: an overweight child perceived as either normal weight (same) or underweight (lighter). On the other hand, overestimation was defined as seeing the child at any higher weight status than he/she really is, for example: an underweight child perceived as either normal weight (same) or overweight (heavier). The perception of a child’s weight status always refers to the maternal perception. Regarding the longitudinal analysis, the outcome was the standardized mean difference (SMD) between BMI at follow-up and at baseline: BMI at follow-up was subtracted from BMI at baseline, and the result was mean standardized (Table 1). BMI was derived from objectively assessed weight and height by trained personnel following standard procedures.

**Table 1:** Definitions of exposure and outcome variables.

Terminology	Definition	Example	Analysis
Perceived…			
…weight status.	Maternal perception of child weight status: *Compared to other children of this age would you say ‘NAME’s’ weight is the same*, *heavier or lighter*?	The same weight, heavier or lighter than other children the same age.	
…health status.	Maternal perception of child health status: *Compared to other children of this age would you say ‘NAME’s’ health is the same*, *better or worse*?	The same, better or worse health than other children the same age.	Exposure in cross-sectional analysis.
Overestimation or underestimation of child weight status.	Combination of maternal perception of child weight status and actual child weight status.	Overweight child (as per BMI) perceived the same weight or lighter than other child the same age.	Outcome in cross-sectional analysis. Exposure in longitudinal analysis.
Actual…			
…child weight status.	Assessed child BMI.	Sex- and age-specific IOTF cut-off points.	The mean standardized difference between follow-up and baseline was the outcome in longitudinal analysis.

#### Exposure variables

For the cross-sectional analysis, the exposure of interest was maternal perception of the child health status. This was assessed with a questionnaire the following question: *Compared to other children of this age would you say ‘NAME’s’ health is the same*, *better or worse*? The perception of a child’s health status always refers to the maternal perception. Regarding the longitudinal analysis, the exposure of interest was underestimation or overestimation of actual child’s weight status as explained above (outcome variables section) (Table 1).

#### Other variables

Other variables included were: child sex, child age (4 years or 5–6 years), weight status of the mother defined as per BMI cut-off points (normal weight, BMI ≥18.5 but <25; overweight, BM ≥25 but <30; obesity, BMI ≥30; and underweight, BMI<18.5), maternal education (none, any school, higher); household location (rural or urban), and household wealth index (bottom, middle and highest). These variables were assessed at baseline (year 2006).

### Statistical analysis

Analyses were conducted using STATA 13 (StataCorp, College Station, TX, USA). For the cross-sectional analysis, first we described the characteristics of the children according to their weight status. Then, we compared actual weight status with perceived weight status, both overall and stratified by maternal perception of their child’s health status. Comparisons between categorical variables were assessed with a Chi-square test. We also used a Kappa test to assess agreement between actual and perceived child weight status. We assessed the strength of the association between maternally perceived child health status (exposure) and underestimation or overestimation of actual child weight status (outcome) using Generalized Linear Models with Poisson family and log-link including robust standard errors. [30] The association estimates are presented as prevalence ratios (PR) and 95% confidence intervals (95% CI). The analyses were conducted using crude and adjusted models, which accounted for child age, sex and BMI, weight status and education of the mother, and household location and wealth index (all assessed at baseline, year 2006). For the longitudinal analysis, the association between underestimation and overestimation of actual child weight status (exposure) with BMI at follow-up (outcome) was assessed using Linear Regression Models, including robust standard errors. Results are presented as the coefficient estimates (β) with 95% CI, and stratified according to maternal perception of child health status. Only adjusted models are presented, and these accounted for: child age, sex and BMI, weight status and education of the mother, and household location and wealth index; all assessed at baseline.

### Ethics

The Young Live Study has ethical approval both generally and in each study setting [31,32]: the Young Lives Study obtained ethical clearance from the Social Science division at University of Oxford; approval in Peru was obtained from the ethics committee of the *Instituto de Investigación Nutricional*. Ethical approval was obtained for the procedures and documents, such as the informed consent. Written informed consent was obtained from everyone involved: children, young people, caregivers and others in the community. All data can be accessed online upon request and it is free of charge: http://www.younglives.org.uk/content/use-our-data.

## Results

### Characteristics of the study population at baseline

Originally, in the Peruvian younger cohort, there were 2,052 children and 1,687 had the questionnaire answered by the mother, of these, 9 children were further excluded because of missing values. Thus 1,678 children were included in the analysis. A total of 846 (50.4%) were boys, and the mean age was 5.3 (SD: 0.4) years. Regarding actual child weight status, 20.2% were overweight while 4.7% were underweight. Table 2 shows the distribution of other variables according to actual child weight status at baseline. Regarding maternal perception of their child’s health status, 32.1% and 7.4% of the children were considered to have better and worse health than their peers, respectively.

**Table 2:** Characteristics of study population according to actual child weight status at baseline.

	Overall (%)	Normal Weight (%)	Overweight (%)	Underweight (%)
Child Sex	N = 1,678	N = 1,261	N = 339	N = 78
Boy	50.4	50.0	53.4	44.9
Girl	49.6	50.0	46.6	55.1
Age	N = 1,678	N = 1,261	N = 339	N = 78
4yrs	20.2	20.7	18.9	18.0
5–6yrs	79.8	79.3	81.1	82.0
Maternal Education*	N = 1,674	N = 1,260	N = 337	N = 77
None	8.1	8.7	5.3	11.7
Any School	72.2	73.7	66.5	71.4
Superior	19.7	17.6	28.2	16.9
Maternal Weight status*	N = 1,666	N = 1,252	N = 337	N = 77
Normal	43.5	47.1	28.8	49.4
Overweight	40.9	39.1	48.7	36.4
Obesity	15.1	13.2	22.6	13.0
Underweight	0.5	0.6	0.0	1.3
Household Location*	N = 1,678	N = 1,261	N = 339	N = 78
Urban	71.3	68.6	78.2	85.9
Rural	28.7	31.4	21.8	14.1
Household Wealth Index*	N = 1,678	N = 1,261	N = 339	N = 78
Bottom	33.9	36.1	27.1	26.9
Middle	32.8	34.3	25.4	39.7
Highest	33.4	29.6	47.5	33.3
Child Health Perception*	N = 1,677	N = 1,260	N = 339	N = 78
Same	60.5	62.0	54.6	62.8
Better	32.1	30.2	39.8	28.2
Worse	7.4	7.8	5.6	9.0

*p<0.05.

### Maternal perception of child weight status at baseline

Over half of the study population (54.1%) was considered to have the same weight as other children the same age; in addition, 17.2% were considered to be heavier. Table 3 depicts the comparisons between actual child weight status versus maternal perception of their child’s weight status, overall and stratified by maternal perception of child health status. Overall, the actual weight of most normal weight children (55.3%) and overweight children (51.3%) were considered to be the same weight as their peers. There was little agreement between actual and perceived weight status: from 8.1% (among children seen with worse health than other the same age) to 11.1% (among children seen as healthy as others the same age).

**Table 3:** Maternal perception and actual weight status of the child, overall and by health status perception.

	Normal Weight (%)	Overweight (%)	Underweight (%)	p-value*	Agreement (%)	Kappa (p-value)
	Overall			<0.001		
	N = 1,261	N = 339	N = 78			
Same	55.3	51.3	46.2		10.4	-0.015 (0.992)
Heavier	13.2	35.4	1.3			
Lighter	31.5	13.3	52.6			
	As Healthy as Others His/her Age			<0.001		
	N = 781	N = 185	N = 49			
Same	60.1	60.5	57.1		11.1	-0.005 (0.736)
Heavier	9.9	26.5	2.0			
Lighter	30.1	13.0	40.8			
	Better Health than Others His/her Age			<0.001		
	N = 381	N = 135	N = 22			
Same	54.1	38.5	31.8		9.7	-0.045 (0.999)
Heavier	22.3	50.4	0.0			
Lighter	23.6	11.1	68.2			
	Worse Health than Others His/her Age			0.009		
	N = 98	N = 19	N = 7			
Same	22.5	52.6	14.3		8.1	0.038 (0.009)
Heavier	5.1	15.8	0.00			
Lighter	72.5	31.6	85.7			

*P-value for chi2 test across groups of weight status.

### Perceived overall child health status and misclassification of child actual weight status: Cross-sectional analysis

Among normal weight children, those perceived as healthier also showed an associated overestimation of their weight status (PR = 2.06, 95% CI: 1.57–2.70); on the other hand, normal weight children perceived as less healthy, were over two times as likely to have their weight status underestimated (PR = 2.17, 95% CI: 1.84–2.55) (Table 4). No significant results were retrieved for underweight children (Table 4). Among overweight children, those perceived as healthier were 36% less likely to have their weight status underestimated (PR = 0.64, 95% CI: 0.51–0.80); however, those perceived as less healthy were almost twice as likely to have their weight status underestimated (PR = 1.78, 95% CI: 1.06–3.00) (Table 4).

**Table 4:** Association between health perception and accurate weight status identification.

	PR (95% CI)		PR (95% CI)	
	Crude	Adjusted	Crude	Adjusted
	**Normal Weight**			
	**Overestimation (heavier)**		**Underestimation (lighter)**	
	N = 864	N = 857	N = 1,093	N = 1,083
As Healthy as Others His/her Age	1	1	1	1
Better Health than Others His/her Age	2.07(1.58–2.72)**	2.06(1.57–2.70)**	0.91(0.75–1.11)	0.93(0.77–1.14)
Worse Health than Others His/her Age	1.31(0.58–2.98)	1.46(0.66–3.21)	2.29(1.96–2.67)**	2.17(1.84–2.55)**
	**Underweight**			
	**Overestimation (same)**		**Overestimation (heavier)**	
	N = 77	N = 75	N = 42	N = -¥
As Healthy as Others His/her Age	1	1	1	1
Better Health than Others His/her Age	0.55(0.28–1.06)	0.51(0.26–1.01)	0.00(0.00–0.00)**	-
Worse Health than Others His/her Age	0.25(0.05–1.55)	0.23(0.04–1.29)	0.00(0.00–0.00)**	-
	**Overweight**			
	**Underestimation (same)**		**Underestimation (lighter)**	
	N = 294	N = 290	N = 165	N = 163
As Healthy as Others His/her Age	1	1	1	1
Better Health than Others His/her Age	0.62(0.50–0.78)**	0.64(0.51–0.80)**	0.55(0.31–0.97)*	0.62(0.36–1.06)
Worse Health than Others His/her Age	1.11(0.81–1.52)	1.16(0.88–1.51)	2.03(1.15–3.58)*	1.78(1.06–3.00)*

Adjusted for child age and BMI at baseline, child sex, maternal educational level at baseline, maternal weight status at baseline, household location and wealth index at baseline. The outcome was overestimation or underestimation of child weight perception. For normal weight children overestimation meant they were regarded as heavier, and underestimation meant they were considered lighter than other children the same age: *Overestimation (heavier)* and *Underestimation (lighter)*, respectively; the same logic applies to underweight and overweight children. When the estimation, as well as the corresponding confidence interval, are zero, it is because the results are rounded up to the nearest hundred; in order words, the estimate effect is very small.

*p<0.05

**p<0.001.

¥Not concave.

### Maternal perception and BMI change: Longitudinal analysis

Mean follow-up time was 2.6 (SD: 0.3) years. No significant results were retrieved with normal weight children; however, there was a trend signaling that normal weight children whose weight status was overestimated tended to gain BMI, while those with their weight status underestimated tended to lose BMI. This pattern held across categories of maternal perception of their child’s health status (Table 5). Overall, underweight children perceived as being the same weight as others of the same age gained more BMI in comparison to underweight children perceived as lighter: β = 0.44 (Table 5). In general, among overweight children, those perceived as lighter (β = -0.87) and of the same (β = -0.55) weight as other, at baseline, lost BMI when compared to those perceived as heavier (Table 5). Similar numbers were retrieved across categories of maternal perception of their child’s health status (Table 5).

**Table 5:** BMI change between baseline and follow-up, overall and according to health perception at baseline.

	Mean BMI Standardized Difference: Follow-up BMI—Baseline BMI					
	Normal Weight		Underweight		Overweight	
	β (95%CI)		β (95%CI)		β (95%CI)	
	**Overall**					
	N = 1,218		N = 75			N = 327
Overestimation (Heavier)	0.09(-0.07; 0.25)	Overestimation (Same)	0.44(0.05; 0.83)*	Underestimation (Same)		-0.55(-0.90; -0.20)*
Underestimation (Lighter)	-0.04(-0.13; 0.05)	Overestimation (Heavier)	0.30(-0.58; 1.18)	Underestimation (Lighter)		-0.87(-1.30; -0.44)**
	**As Healthy as Others His/her Age**					
	N = 753		N = 48		N = 180	
Overestimation (Heavier)	0.05(-0.16; 0.25)	Overestimation (Same)	0.47(-0.15; 1.10)	Underestimation (Same)	-0.71(-1.17; -0.24)*	
Underestimation (Lighter)	-0.04(-0.16; 0.07)	Overestimation (Heavier)	0.35(-0.91; 1.62)	Underestimation (Lighter)	-1.25(-1.84; -0.66)**	
	**Better Health than Others His/her Age**					
	N = 370		N = 21		N = 129	
Overestimation (Heavier)	0.10(-0.15; 0.35)	Overestimation (Same)	0.61(-0.51; 1.72)	Underestimation (Same)	-0.39(-0.96; 0.19)	
Underestimation (Lighter)	-0.01(-0.21; 0.20)	Overestimation (Heavier)	Omitted	Underestimation (Lighter)	-0.25(-1.01; 0.50)	
	**Worse Health than Others His/her Age**					
	N = 94		N = 6		N = 18	
Overestimation (Heavier)	-0.19(-1.00; 0.62)	Overestimation (Same)	Omitted	Underestimation (Same)	-1.64(-3.78; 0.49)	
Underestimation (Lighter)	0.10(-0.24; 0.44)	Overestimation (Heavier)	Omitted	Underestimation (Lighter)	-1.76(-3.66; 0.14)	

Only adjusted models are presented, these included: child age and BMI at baseline, child sex, maternal educational level at baseline, maternal weight status at baseline, household location and wealth index at baseline. The exposure variable was overestimation or underestimation of child weight status; for normal weight children overestimation meant they were regarded at heaver, while underestimation meant they were regarded as lighter than other children the same age: *Overestimation (heavier)* and *Underestimation (lighter)*, respectively; the same logic applies to underweight and overweight children. Accurate identification of weight status was the reference category in all models. Interpretation of results: a coefficient equals to 0.22 means there has been a BMI increase equals to 0.22 Standard Deviation; if coefficients are negative, there has been a BMI decrease.

*p<0.05

**p<0.001.

## Discussion

### Main findings

In a sample of children living in resource limited settings in Peru, there is a lack of agreement between actual and maternal perception of child weight status: overweight children are often perceived as lighter than their actual weight. Overestimation and underestimation of actual weight status was influenced by maternal perception of their child’s health status: normal weight children considered to be healthier than their peers were more likely to have their weight overestimated; yet normal weight children considered to be less healthy were more likely to have their weight underestimated. Such perceived overestimation or underestimation of a child’s actual weight seems to influence the child’s future BMI. Among overweight children, those perceived as of the same weight or lighter than their peers showed a greater loss of BMI points compared to overweight children who were perceived as heavier.

### Comparison with previous studies

Although most previous reports have been conducted in developed countries, their results agree with ours: weight underestimation of overweight children is frequent [6,8,12–15]. Previous studies have focused on socioeconomic variables as determinants of the (mis)perception of child weight status: it was more likely for younger children or children with overweight mothers to have their weight status inaccurately perceived. This study, on the other hand, focuses on a single possible determinant—maternal perception of their child’s health status—which had not been much explored, neither in developing countries where cultural profiles are more likely to influence perception of childhood weight and health status [17,20–24].

Other studies have assessed the long-term weight changes of (in)accurate perception of actual child weight status [33,34]. Children whose weight was underestimated early in childhood gained more weight, and yet those whose weight was overestimated lost more weight, as assessed at age 7 years [33]. Gerards et al. reported that among 5-year-old overweight children, those who had their weight status accurately perceived, versus their peers inaccurately perceived, had a greater increase in BMI z-score at age 6, 7, 8 and 9 years; similar to our results, they found that overweight children, those whose weight was underestimated had a greater BMI loss than their peers whose weight was accurately estimated [34]. Other studies of adolescents showed that body size underestimation was associated with a lower risk of being overweight [35]. On the other hand, youth who thought of themselves as overweight had a greater risk of becoming obese [36]. Of note, these studies were conducted in developed countries limiting the extrapolation of their results to our study population.

### Interpretations of results

The agreement between perceived and actual childhood weight status is very low, and agreement varied according to maternal perception of their child’s health status. We hypothesized that weight status overestimation would be more likely in children perceived to be more healthy; conversely, that weight status underestimation would be more likely in children perceived to be less healthy. This scenario is clearly depicted for normal weight and overweight children. There seems to be a subjective equivalence between healthy and “chubby,” as well as between “chubby” and good parenting [16–18,20–24]. A study compared Mexican mothers living in Mexico and in the USA, and found that the former wanted their children to be bigger [18]. Moreover, foreign-born Hispanic mothers with less acculturation do not see childhood obesity as unhealthy [17,20–24]. Our results further support the strong association between maternal perception of their child’s health and weight status in a resource-limited setting in South America. This implies that pediatricians and other health professionals should work with mothers to improve their subjective perception of their child’s weight and health status.

When assessing future weight according to underestimation or overestimation by stratified levels of maternal perception of child health status, many of our results did not show a significant association. This could be explained by lack of statistical power. This issue could be resolved by other prospective studies that include a larger sample size, or one with higher prevalence of overweight children. Nevertheless, many of the results for overweight children did show significant trends. All overweight children lost BMI regardless of the maternal perception of their health status. This suggests that maternal perception of their child’s health status does not influence whether the overweight child loses or gains weight, but influences the magnitude of such change. On the other hand, overall, underweight children perceived to be of normal weight, gained more BMI than underweight children whose weight was accurately identified. Since underweight children would have to gain much weight to reach obesity, their parents should be taught about the importance of keeping a normal weight status so they would not go from underweight to overweight or obesity. In general, misperception in overweight children seems to be beneficial, because it would make them lose more weight, than if they had been accurately identified; also, overestimating seems to be beneficial for underweight children, because it makes them gain more weight, than if they had been accurately identified. A possible explanation is the age-range of the study population. Children had an average age of 5.3 years at baseline and 7.9 years at follow-up. In this age-range children start school and engaging in sports activities. This could explain the weight loss among overweight children, as well as the minor weight gain for underweight children.

The results for normal weight children seem counter-intuitive: we expected that normal weight children perceived as heavier would lose weight, though those perceived as lighter would gain weight. One possible explanation is that parental perception is determined by various cultural-psycho-social features that could influence a child to gain or lose weight, such as parental willingness to change their child’s weight status [37], or parental dietary profiles [38,39]. Because these results did not show significant associations, they should be verified by future studies.

### Strengths and limitations

This study included a large sample size of children living in resource-limited urban and rural settings in Peru. Further, this study’s use of prospective data is unique. Nevertheless, limitations of the study must be highlighted. First, the study sample was not representative of the country; yet it provides information regarding children in a developing country. Other studies in developing countries are needed, including explanatory variables of parental acculturation and dietary profiles. Second, the assessment of perceived child weight status was conducted with a questionnaire. Other instruments have been developed and some include figures to make comparisons. Although it has been suggested that the use of image scales improve the accurate recognition of overweight children, it is not enough to draw a definitive conclusion of its superiority: studies have compared different assessment tools and reported no difference in (mis)perception [40]. Third, the precise question used in this study could have added response bias. Mothers were asked to compare their children to other children of the same age. If the proportion of overweight children was high, taking into account that overweight children tend to cluster, mothers could have perceived their children to be the same weight as other overweight children. This should not have affected our results because only 20% of children in our sample population were overweight (includes obesity) and mean BMI at baseline was 16.4 (±1.8) (normal range). Fourth, regarding the results of actual weight status by perceived weight status according to each level of health status, some of the cells in the corresponding table included a small sample. These results should be interpreted with caution.

### Conclusions

There is poor agreement between actual and maternal perception of their child’s weight status. Overestimation or underestimation of child weight status seems to be influenced by maternal perception of child health status. Such (mis)perception of weight status might influence future BMI.

## Supporting information

S1 File Maternal perception & BMI [complete no dates]. This file contains data analyzed for this study.
(DTA)
